# The internal organizational performance influence factors study-an empirical test

**DOI:** 10.1371/journal.pone.0298595

**Published:** 2024-04-04

**Authors:** Xiuling Yuan, Lihua Ma, Cheng Wang, Huizhe Yan, Yufei Chen

**Affiliations:** 1 School of Management Engineering and Business, Hebei University of Engineering, Handan, 056009, China; 2 Faculty of Business, Department of Management Engineering, Nanfang College Guangzhou, Guangzhou, 510970, China; 3 Uibe Business School, University of International Business and Economics, Beijing, 100105, China; University of Cape Coast, GHANA

## Abstract

With the changes of social and economic development, more and more people pay attention to the development of non-profit organizations, and the performance research of non-profit organizations has become the focus of research. As the internal governance organization of non-profit organization, the board of directors and the management organization are related internal factors that will affect the organizational performance of non-profit organization. Based on the data of Form 990 of the US Internal Revenue Service, this paper conducted an empirical study on the relationship between internal governance and organizational performance of non-profit organizations, and studied the moderating effects of board size, average weekly working hours, number of managers, members’ work involvement and compensation incentives on internal governance and organizational performance of non-profit organizations. The results show that the number of managers in non-profit organizations is negatively correlated with organizational performance, the average weekly working hours of managers are significantly correlated with organizational performance, and the compensation of managers is significantly correlated with organizational performance. Through the empirical demonstration, this study promotes the management and development practice of non-profit organizations, and lays a solid foundation for the construction of socialist harmonious society in China.

## 1.Introduction

Non-profit organizations are becoming more and more crucial in modern society for advancing social progress and resolving social issues. However, non-profit organizations internal governance and organizational performance issues have gained more attention as a result of its ongoing growth in size and service area. The non-profit organizations internal governance system is essential to its performance and operation, and the organization’s capacity for sustainable development and social impact are closely correlated with its performance level.

An organization’s ability to function is essential to its growth. The organizational performance measuring system in profit organizations is progressively enhanced. Jardioui pointed out the impact of internal governance culture on organizational performance, and the research on organizational performance was further developed [1]. Wang conducted an empirical study on organizational performance based on the internal governance environment of non-profit organizations, and highlighted the impact on organizational performance [2]. Harrison indicates that organizational performance plays a key role in the important decision-making of managers’ transformation development [3].

A new investigation by Sutharshini, Thevanes, and Arulrajah examined the effect of managing time on the effectiveness of organizations. After decades of development, the performance evaluation system of for-profit organizations is relatively perfect [4]. However, for non-profit organizations, there is still a big gap in the research on the organizational performance of non-profit organizations. Some researchers have also conducted some studies on the performance of non-profit organizations. For example, Al-dmour studied the correlation between corporate performance of non-profit organizations and corporate marketing [5]; Ortiz J.I.B uses the DART model to analyze the performance impact of non-profit organizations and provide effective suggestions for managers and decision makers [6]; Oliveira et al. studied the defects of knowledge sharing to improve organizational performance by establishing a matrix of human capital appreciation and performance in non-profit organizations [7].

In comparison to for-profit businesses, the study on the organizational performance of non-profit organizations is still lacking, despite the fact that several scholars have examined this topic. The research was conducted due to the significant differences between for-profit and non-profit organizations regarding their techniques, development goals, and sources of income. For-profit businesses mostly depend on stock investors’ investments in the stock market as their primary source of funding. As a result, for-profit businesses must focus more on profitability. Government funding and gifts from other businesses and individuals are the two primary sources of income for nonprofits. When it comes to development, the goal of for-profit businesses is to increase earnings through investment and market operation. Public welfare is used by nonprofit organizations for development, service, academic, philanthropic, and environmental protection goals. As a result, evaluating the organizational effectiveness of non-profit organizations is very challenging [8].

The performance of non-profit organizations is becoming more and more significant as the government and public pay greater attention to them. Because of their unique characteristics, non-profit organizations may face issues during their development, including inefficiency, waste, and corruption. These issues are typically brought on by the managers of non-profit organizations’ lack of foresight [9]. LeClair showed that the malfeasance in non-profit organizations would lead to soft corruption [10]. BouChabke believes that the phenomenon of corruption in non-profit organizations is very serious, such as chaotic organizational environmental governance and low management efficiency [11]. Timely intervention and adjustment should be made to provide good suggestions for managers. Francisco non-profit organizations lack of social attention to organizations and lack of relevant manager training, resulting in a relatively low organizational performance [12]. Outside of nonprofits, those who donates, grants, contracts, and volunteer time to non-profits tend to evaluate non-profits’ performance with relatively simple financial information, which they consider "overhead." This is better for companies that spend less than "administrative costs." This is unfair to the managers of non-profit organizations. The idea of using spending to evaluate nonprofits’ performance forced managers to cut organizational spending and lower executive pay, but it also created corruption and inefficiency.

Khorin A. N indicates that non-profit organizations are facing serious financial crisis, economic crisis, efficiency crisis and legitimacy crisis [13]. Therefore, the research on the relationship between internal governance and organizational performance of non-profit organization is of great significance for the future development of non-profit organization. In terms of organizational performance, Pejcal believes that the financial disclosure of non-profit organizations is crucial to the performance evaluation of non-profit organizations [14]. Based on the Czech Foundation, he conducted research on relevant organizations and the specific situation of land and earth. Lopez-Cabrera pointed out the job analysis of employee satisfaction, pointed out the unsatisfactory aspects of performance, and provided directions for human resource management and performance improvement of non-profit organizations [15]. Based on previous studies, Mushafiq summarized the relationship between financial performance indicators and return on assets [16]. In terms of internal governance, Kuroki points out that the board of directors has an important influence on non-profit organizations and will play a decisive role in internal governance [17]. Among them, the time, skills and experience of the board of directors have an impact on the effectiveness of the board of directors of non-profit organizations. The Board and management share a common vision for the governance of non-profit organizations. Prouteau believes that the key to improving the performance of non-profit organizations is that the board of directors should change and measure the relationship between the organization and financial resources and other issues [18]. Bradford et al. believe that governance plays an important role in non-profit organizations, and the level of time and energy invested by board members will also directly affect the quality of the board’s decision-making and supervision work, thus affecting the performance level of the organization [19]. Roshayani A shows that non-profit boards play a key role in ensuring that they are properly managed. Non-profit organizations need a strong and active board of directors to perform some form of internal oversight to help the organization achieve better performance [20].

The purpose of this paper is as follows: (i) To study the incentive effect of board size, member input, weekly working hours and salary rewards on organizational governance and performance; (ii)Analyze the moderating role of compensation incentives in the internal governance and organizational performance of non-profit organizations, especially in improving the performance of the board of directors and management; (iii) Make relevant recommendations to promote the internal governance and organizational performance optimization of non-profit organization, and provide practical guidance for relevant decision makers and managers.

In order to better understand the moderating role that pay incentives (management expenditures) have on internal oversight and organizational performance of non-profit organizations, this research performed an empirical investigation on the link between internal governance and organizational performance of non-profit organizations. This study is significant as follows: (i) adds to the body of relevant empirical research on nonprofit organizations; (ii) enhances the criteria used in prior studies to evaluate organizational performance; (iii) advances the field’s understanding of the relationship between non-profit organizations and organizational performance.

## 2.The theory and hypothesis

### 2.1 Board size

The group of directors, which forms the backbone of the non-profit organization, is the highest-level governing body in the whole, setting the organization’s mission and goals and serving as a conduit between it and the outside world. The non-profit organization’s Board of Directors oversees the yearly budget, final accounting, and day-to-day operations in addition to soliciting cash for the organization. Aggarwal, Evans, and Nanda examined 990 tabular data points from three non-profit organizations between 1998 and 2003 and discovered a significant correlation between the non-profit organizations’ performance and the number of their board of directors [21]. Zhu, through a survey of 217 for-profit organizations and 156 non-profit organizations in Canada, shows that different processes lead to different levels of strategic participation by the board of directors, and this impact depends on the type of organization concerned [22]. In addition, boards that are actively involved in strategic decisions can improve the performance of their organizations. Roshayani A shows that in these organizations, non-profit organization boards play a key role in ensuring that they are properly managed [20].

It is challenging for donors overseeing non-profit organizations to control behavior within the organizations. Internal supervision is essential to non-profits. A larger board aids in keeping an eye on management infractions and lessens organizational shortsightedness. According to Lambert, there is a favorable correlation between the growth of an organization’s board size and improved organizational performance in non-profit organizations [23]. The advent of management and the growth of the board of directors would successfully curb the incidence of managerial corruption and inefficiencies. The analysis presented above leads this study to suggest the following theories:

**Hypothesis 1**: Board size is positively correlated with the performance of non-profit organizations

### 2.2 Work input of board members

For non-profit boards, engaging board members in their work can be a very challenging task. First off, there is no compensation for non-profit board members. These individuals join non-profit groups in order to further the common good. In addition to providing donations to charitable organizations, they also require volunteer labor. This is an attempt to gauge these board members’ loyalty. Furthermore, board members are not employed full-time. These board members run their own companies, and the board has a lot of work ahead of it both a for-profit entity and a crucial conduit for outside communication. These directors are essential to non-profit organizations as they manage boards, check documentation, and raise money. For instance, Brunetto shows that board members of non-profit organizations are more involved in the social growth of the organizations and the services they offer, as well as having a high level of work involvement in the actual management process [24]. Through the board of directors of non-profit organizations, Treinta supports organizations with financial communication and regulatory work, thereby promoting the enhancement of non-profit organizations’ performance [25].

The board is not a simple position. The long-term growth of the non-profit organization and the accomplishment of the council’s duties are significantly impacted by the non-profit council’s activities. When the non-profit board members’ average working hours are high, it means that the organization has a strong sense of cohesiveness and an effective management system, and that the board members can accomplish more work. The analysis presented above leads this study to propose the following hypotheses:

**Hypothesis 2:** Board members’ work input is positively correlated with the performance of non-profit organizations

### 2.3 Number of managers

Previous research focused more on the significance of the board of directors than it did on the function of managers in non-profit organizations. In actuality, managers are paid by non-profit organizations. Greater funding for charitable organizations and more transparent work distribution. High compensation levels are enjoyed by managers at non-profit institutions. Rosnerova, adding more managers will exacerbate the lag impact on non-profit organizations’ management and decision-making processes [26]. Wicker, the rise of managers within non-profit organizations will result in a hierarchy of leaders, and the disparity in decision-making will impair these organizations’ ability to function as an organizational unit [27]. When it comes to management size, performance can be enhanced by adding more managers within a specific range. However, we generally think that a non-profit’s high level of efficiency is not correlated with its big number of managers. Conversely, having a lot of managers results in a lot of duplicate individuals and organizations, which lowers management effectiveness and raises expenses. Based on the above analysis, this study proposes the following assumptions:

**Hypothesis 3**: The number of managers is negatively correlated with the performance of non-profit organizations

### 2.4 Average weekly working hours of managers

Kramer W. and others To improve the internal management of businesses and achieve greater company development, managers should implement change management [28]. Financial, human, and other resources are used by managers to complete tasks and move the company closer to its goal. Managers must so continue to be proactive and committed to the goals and objectives of the non-profit. In the event that the management is largely inactive, non-profits will be favored. This will make it possible for opportunism to take the place of planning, diversification, or even ineffectual management members’ efforts. This calls on managers to take an active role in the job, and managers’ active involvement is beneficial to the growth of non-profit organizations. Positive changes in managers’ job engagement are expected to positively impact organizational performance as well. Managers’ work dedication can be objectively reflected in their average weekly working hours. The analysis presented above leads this study to propose the following hypotheses:

Hypothesis 4: Managers’ average weekly working hours are positively correlated with the performance of non-profit organizations

### 2.5 Salary incentives

For a very long time, researchers have been studying CEO compensation. But for-profit companies have been the subject of the majority of research. The usefulness of pay incentives in non-profit organizations has not received much research. Over the past 20 years, NGOs have experienced remarkable growth, but there hasn’t been much effort put into figuring out how effective their manager incentives and compensation are. It’s even a common belief that non-profit managers shouldn’t get compensation. Dai, there is a considerable positive correlation between the income from donations received by non-profit organizations and CEO salary incentives [29]. According to another studies, executives are more likely to enhance information transparency in order to increase donation money when their organizations perform well; in other words, their intermediary effect is more substantial. Non-profit organizations are not subject to the pressure and limitations that for-profit firms’ shareholders put on them, in contrast to for-profit organizations. Compared to compensation plans provided by for-profit companies, even non-profit pay plans usually have higher institution-specific requirements and lower base incomes. Non-profits, however, continue to get a lot of attention as they may hire more competent managers for less money and because their managers are frequently less content. In the view of Ramirez et al, there may be a hierarchical implementation of the compensation system and non-profit organization pay cannot achieve the incentive effect within the traditional economic system [30]. While social mission and personal values serve as the main motivators in non-profit organizations, managers’ and employees’ commitment to organizational goals can be strengthened with the right remuneration incentives, which will increase internal governance effectiveness and organizational performance.

In particular, an effective compensation plan may encourage managers to supervise project execution, manage resources more effectively, and make wiser strategic choices that further the organization’s goals. Overall, their findings demonstrate that financial incentives are positively connected with non-profit performance and that non-profit managers are rewarded for their contributions to the advancement of organizational goals. Pay incentives have a significant impact on organizational performance in both for-profit and non-profit organizations. Nonetheless, we think that the influence of internal governance on organizational performance is moderated in non-profit companies by remuneration incentives. Based on the above analysis, this study proposes the following assumptions:

Hypothesis 5: Salary incentives play a regulatory role between internal governance and corporate performance of non-profit organizations

## 3. Research process

### 3.1 Data sources

The data for this study was derived from the IRS (Internal Revenue Service) Form 990. We selected data from 20 non-profit organizations in 2017. These research institutions include KAISER FOUNDATION HEALTH PLAN INC, YALE UNIVERSITY, NEW YORK STATE CATHOLIC HEALTH PLAN INC, INOVA HEALTH CARE SERVICES, and the like. We chose Form 990 as the data source because the US Internal Revenue Service redesigned the 990 form in 2007 to reinforce the regulation of non-profit organizations in order to solve the problem of low disclosure of non-profit organizations. The new 990 form increases the amount of data that non-profit organizations need to fill out. These data are highly transparent and accurate. At the same time, the data in the 990 form is also very comprehensive, which can fully reflect the internal governance and organizational performance of non-profit organizations. We selected five kinds of data that reflect the internal governance of non-profit organizations, and combined with the previous research to develop 11 indicators that can reflect the performance of non-profit organizations.

### 3.2 Performance indicators of non-profit organizations

Jardioui, LeClair, and Pejcal selected three indicators and added eight more that indicate the performance of non-profit organizations in their research findings [1,10,14]. Investment revenue currently makes up a sizable portion of non-profit organizations’ income structure because investment indicators were rarely included in earlier research. Consequently, two investment indicators are included to this article, particularly "The proportion of unrealized income from investment to total income," which highlights the unsatisfactory aspect of non-profit organizations’ performance. The following summarizes the general state of non-profit organizations’ performance indicators: The first indicator shows the percentage of net income to total revenue and is calculated by dividing total revenue minus total expenses by total revenue; The second indicator represents net profit as a proportion of total assets and is calculated by dividing total revenue less total expenses. The third indicator shows the percentage of net assets to total assets by dividing net assets (fund balance) by total assets; The logarithm of total revenue, base 10, is shown in indicator 4; the logarithm of donation income, base 10, is shown in indicator 5; and the ratio of donation income to total income, divided by total income, is shown in indicator 6. The logarithm of welfare spending, based on 10, is shown in indicator 7; The welfare expenditure to total assets ratio is shown in Indicator 9; the welfare expenditure to total income is shown in Indicator 8; and the investment income to total income is shown in Indicator 10, which is the investment income divided by total income and expressed as a percentage of total income. As a proportion of total income, the unrealized investment return is represented by Indicator 11, which is calculated by dividing it by the total revenue.

### 3.3 Research design

The study is divided into two phases. In the first stage, we designed eleven indicators that reflect the performance of non-profit organizations based on previous research, and standardized the data needed in the indicators. Then use the factor analysis method to reduce the dimension of the performance of the non-profit organization, and hope to find the common factor that can reflect the performance of the non-profit organization, and calculate the common factor score, on the basis of which the common factor comprehensive score is calculated. In the second stage, the correlation analysis of five variables reflecting the internal governance of non-profit organization was carried out to determine the degree of correlation. In the third stage, based on the relevant analysis, the regression analysis is carried out on the size of the board of directors and the weekly working hours of the board members to test the variable adjustment effect of the corresponding salary reward.

### 3.4 Research method

This paper uses factor analysis method to analyze the relationship between the number of managers, average weekly working hours, salary and other variables and the performance of non-profit organizations, which is an efficient method. First of all, the relevant data is collected, including variable data of internal governance and organizational performance of non-profit organization, to ensure that the data meets the basic assumptions of factor analysis, such as the correlation between variables and the size of the sample. Secondly, the collected variable data is input into the statistical software and the factor analysis method is selected. In this paper, principal component analysis is selected for factor extraction. In this step, the number of potential factors and extraction factors are determined. Once potential factors are extracted, the relationship between factors can be better explained by variance maximum rotation method. Finally, by analyzing the output results of the statistical software, the factor load matrix after rotation is interpreted to understand the meaning of each potential factor representation. Based on the results of factor analysis and rotation maximum variance method, conclusions are drawn and suggestions are put forward to guide the improvement of internal governance of non-profit organizations and improve organizational performance.

This paper adopts correlation analysis and causation analysis. Correlation analysis is a statistical analysis method to study the correlation between two or more random variables in the same position. For example, between a person’s height and weight; The correlation between the relative humidity in the air and the rainfall is the problem of correlation analysis. Causal analysis shows that one event is the result of another event; In other words, there is a causal relationship between the two events. Regression analysis is a kind of causal analysis, this question adopts regression analysis method. The difference between correlation analysis and regression analysis: Regression analysis focuses on the study of the dependence between random variables in order to use one variable to predict another variable; Correlation analysis focuses on the discovery of various correlation properties between random variables. Correlation analysis is the analysis of signs that are indeed connected in the population, and its main body is the analysis of signs that have causality in the population. It is the process of describing the closeness of the relationship between objective things and expressing it with appropriate statistical indicators.

## 4 Results and discussion

### 4.1 Factor analysis

In this paper, SPSS 25.0 software is used to standardize the performance indicators of 11 non-profit organizations, and factor analysis method is used to rotate maximum variance. According to the eigenvalue is greater than 1, the common factor is extracted, and the factor analysis is carried out on 11 indexes. Using KMO test and Bartlett sphericity test, we found that the KMO value of these 11 indicators was 0.485 and the Sig value was 0(KMO>0 is usually required). Bartlett sphericity test Sig≤0.05 can be used for factor analysis.) Obviously, some of the 11 indicators are not sufficiently correlated with other variables, and they need to be tested to exclude indicators with insufficient correlation. Therefore, we combined these 11 indicators in order, conducted KMO test and Bartlett sphericity test, and selected the combination with the largest KMO value. It is found that the KMO value of the 9 indicators except "total income minus total expenses" and "donation income" is the largest, with KMO value reaching 0.619 and Sig value being 0, which is consistent with the result of factor analysis.

Table 1 showed the common factor eigenvalues and contribution rates. From the results of factor analysis, the total variance explanation rate of the 9 indicators is 74.032% (usually greater than 70%). This shows that the information loss of the original explanatory variables is less, and the result of factor analysis is satisfactory. The eigenvalues of the three factors are all greater than 1, which are 3.029, 1.745 and 1.634, respectively. According to the change trend of gravel map, the first three factors change greatly. Starting with the fourth factor, the eigenvalues tend to stabilize. This result suggests that we should extract three common factors. At the same time, the cumulative contribution rate of these three common factors is 74.032%, indicating that these three common factors can better evaluate these nine performance indicators.

**Table 1:** Common factor eigenvalues and contribution rates.

	EIGENVALUES	ROTATING LOAD	CUMULATIVE CONTRIBUTION RATE
**COMMON FACTOR 1**	3.029	32.365%	32.362%
**COMMON FACTOR 2**	1.745	20.485%	54.159%
**COMMON FACTOR 3**	1.634	24.325%	74.032%

Table 2 showed the factor load and factor score load. In factor analysis, the results of the initial factor load matrix after the maximum variance orthogonal rotation are as follows.

**Table 2:** Factor load and factor score load.

INDICATOR NAME	FACTOR LOAD			FACTOR SCORE LOAD		
	Common factor 1	Common factor 2	Common factor 3	Common factor 1	Common factor 2	Common factor 3
WELFARE EXPENDITURE DIVIDED BY TOTAL INCOME	0.902	0.185	-0.145	-0.068	0.485	-0.145
WELFARE EXPENDITURE DIVIDED BY TOTAL ASSETS	0.988	-0.265	-0.185	0.054	0.005	-0.485
DONATION INCOME DIVIDED BY TOTAL INCOME	0.845	-0.057	0.006	0.035	-0.146	0.396
WELFARE EXPENDITURE	0.516	0.281	0.475	0.295	-0.052	0.023
UNREALIZED GAIN ON INVESTMENT DIVIDED BY TOTAL INCOME	0.193	0.852	-0.059	0.216	0.198	0.275
(TOTAL REVENUE MINUS TOTAL EXPENSES) DIVIDED BY TOTAL REVENUE	-0.175	0.802	-0.281	0.375	0.085	-0.075
INVESTMENT INCOME DIVIDED BY TOTAL INCOME	0.002	0.588	0.553	0.302	-0.153	-0.075
NET ASSETS (FUND BALANCES) DIVIDED BY TOTAL ASSETS	0.285	0.057	-0.715	-0.005	0.329	0.345
TOTAL REVENUE	0.046	-0.236	0.712	0.0652	0.488	-0.008

Table 2 shows that the first common factor consists of four variables: “welfare income divided by total assets”, “welfare consumption divided by total income”, and “welfare income divided by total income”. With an average load of 84.3%, it is high. These four factors show how well non-profit organizations do in carrying out their welfare obligations as well as their income level from welfare. As a result, “Welfare performance” was our choice for common factor 1. The average load of the second component is higher for the three variables: “unrealized investment income divided by total income,” “(total income minus total expenses) divided by total income,” and “investment income divided by total income.”. The average is 75.8%. The profitability of non-profit organizations is represented by these three variables, which also reflect it. Consequently, we used the term “Profitability” for common factor 2. The variable “total income” has a greater average burden for the third element, averaging 74.2%. This variable measures the non-profit organization’s revenue as well as its overall financial health. As a result, we gave the term “financial situation” to common factor 3. The following are the specifics of the three shared factors: Common factors 1 and 2 are welfare performance, which shows that the non-profit receives internal and external donations; profitability, which indicates the non-profit organization’s profitability; and financial condition, which expresses the non-profit organization’s financial condition.

On this basis, by calculating the scores of the three common factors, the score of the comprehensive performance index Y of non-profit organizations is calculated. Y = (32.365 * Principal component C1 score + 20.485 * principal component C2 score + 24.325 * principal component C3 score)/ 74.032.

### 4.2 Descriptive statistical analysis

Based on the assumptions, we selected six indicators from Form 990 and performed descriptive statistical analysis, as was shown in Table 3. The results are as follows.

**Table 3:** Descriptive statistical analysis results.

VARIABLE NAME	MIN	MAX	M	SD	V
**1.NUMBER OF DIRECTORS**	9	505	81.25	127.419	15788.25
**2.AVERAGE WEEKLY WORKING HOURS OF DIRECTORS**	2	48.6	10.956	12.846	152.419
**3.NUMBER OF MANAGERS**	2	202	34.1	52.006	2695.236
**4.AVERAGE WEEKLY WORKING HOURS OF MANAGERS**	11.35	67.55	35.264	16.416	325.718
**5.NUMBER OF MAJOR MEMBERS OF THE ORGANIZATION**	25	616	131.25	159.58	25668.416
**6.MANAGERIAL COMPENSATION**	1	8.045	6.748	1.745	2.698

According to descriptive statistical analysis, we can see that the 20 non-profit organizations have a very large gap in the number of directors, the number of managers and the number of major members of the organization, reflecting the current large gap between the non-profit organizations in the United States. Especially in the number of directors, the maximum number of directors reached 505, and the minimum value was only 9 people. In addition, we can see that the average weekly working hours of directors is very low. This data indicates that directors rarely participate in the work of non-profit organizations, and the current board of directors still lacks supervision of non-profit organizations. The minimum salary of management personnel is 0, indicating that some non-profit organizations not only have unpaid work on the board, but also managers have no pay.

### 4.3 Correlation analysis

Table 4 showed the correlation analysis. Based on the results of the correlation analysis, we found that the number of directors is negatively correlated with the overall performance score of the organization, but it is not significant, and it is impossible to prove hypothesis one (the size of the board of directors is positively related to the performance of non-profit organizations). According to our previous assumptions, a company with Large independent boards of many independent directors can better supervise the managers of non-profit organizations, thereby enabling better organizational performance. The data shows that the relationship between the size of the board of non-profit organizations and the organizational performance of non-profit organizations is not significant, which indicates that the directors of non-profit organizations may have failed to effectively perform management and supervision functions. Directors’ failure to give full play to management and supervision functions may be due to the nature of non-profit organizations. Due to the non-profit characteristics of non-profit organizations, the organizational performance of non-profit organizations is generally not related to the interests of directors. Therefore, it is difficult for directors of non-profit organizations to truly play their role as directors, which has led to the failure of board governance of non-profit organizations. In addition, some members of the board of directors take advantage of the defects in the articles of association of the non-profit organization to restrict external members from joining the board of directors, and thus jointly control the board of directors through related parties. This also affects the effectiveness of the board of directors; the average weekly working hours of directors is not significantly related to the overall score of the organization’s performance, and it is impossible to prove hypothesis two (the work input of board members is positively related to the performance of non-profit organizations), which indicates the work of directors The degree of investment does not directly affect organizational performance, it can only play an indirect role—influencing organizational performance through supervision and management of managers; the number of managers is significantly negatively correlated with the overall score of organizational performance, so assumption three (the number of managers and non-profit Organizational performance is negatively correlated). Validated our speculation that too many managers can cause inefficiencies and reduce organizational performance. The compensation of managers is significantly related to the comprehensive score of organizational performance, indicating that the compensation of managers will significantly affect the performance of non-profit organizations. It is possible to test the effectiveness of regulation. In addition, the relevant analysis results show that the number of directors is significantly correlated with the number of managers, indicating that the size of non-profit organizations determines both the number of directors and the number of managers. The number of managers is negatively correlated with the average hours worked per week, indicating that an increase in management reduces the average hours worked per manager.

**Table 4:** Correlation analysis.

VARIABLE NAME	1	2	3	4	5	6	7
**1.NUMBER OF DIRECTORS**	1						
**2.AVERAGE WEEKLY WORKING HOURS OF DIRECTORS**	0.052	1					
**3.NUMBER OF MANAGERS**	0.945**	0.196	1				
**4.AVERAGE WEEKLY WORKING HOURS OF MANAGERS**	-0.452*	-0.145	-0.506*	1			
**5.NUMBER OF MAJOR MEMBERS OF THE ORGANIZATION**	0.9025**	0.372	0.9115**	-0.552*	1		
**6.MANAGERIAL COMPENSATION**	0.284	0.288	0.306	-0.043	0.315	1	
**7.ORGANIZATIONAL PERFORMANCE COMPREHENSIVE SCORE**	-0.076	0.081	-0.045	0.4855**	0.034	0.341*	1

Note: * means p < 0. 05, ** means p < 0. 01.

Based on the results of the correlation analysis, we conducted a regression analysis to verify the weekly working hours to see if there was a significant relationship. Then, after determining the relevant variables, we test whether the compensation incentive has a regulating effect on the internal governance and organizational performance of non-profit organization.

### 4.4 Regression analysis

On the basis of relevant analysis, model regression analysis was carried out to verify the above research hypotheses. Specifically, weekly working hours were incorporated into the model for regression analysis. This paper takes organizational performance as the dependent variable, board size, number of managers and management compensation as the control variables. The results are shown in Table 5.

**Table 5:** Regression analysis result.

VARIABLE	ORGANIZATIONAL PERFORMANCE	
**CONTROL VARIABLE**		
**NUMBER OF DIRECTORS**	**0.201**	**-0.0152**
**NUMBER OF MANAGERS**	**-0.312**	**0.149** *
**NUMBER OF KEY MEMBERS OF THE ORGANIZATION**	**-0.268**	**-0.034**
**MANAGEMENT COMPENSATION**	**0.302**	
**INDEPENDENT VARIABLE**		
**MANAGEMENT WEEKLY WORKING HOURS**	0.163***	
**AVERAGE WEEKLY WORKING HOURS OF DIRECTORS**		0.236***
**R** ^**2**^	**0.054**	**0.075**
**ΔR** ^**2**^	**0.052**	**0.051**
**F-NUMBER**	**2.036** *	**3.415** ***

Note: * means p < 0. 05, ** means p < 0. 01.

The regression results show that the regression coefficient of directors’ weekly working hours on organizational performance is 0.163, and the significance p < 0.001, that is, directors’ weekly working hours have a significant positive impact on organizational scale. The regression coefficient of the average weekly working hours of directors on organizational performance is 0.236, and the significance p < 0.001, that is, the working hours of directors have a significant positive impact on organizational performance. The average weekly working hours of managers are significantly correlated with organizational performance, which can prove the validity of hypothesis four (the weekly working hours of managers are positively correlated with the performance of non-profit organizations). As the core of non-profit organization operation, managers play an important role in organizational performance. When the average working hours of non-profit organizations increase, it will lead to positive changes in organizational performance.

### 4.5 Adjustment effect test

The adjustment effect test is to study whether the effect of the weekly working hours of the management staff on the performance of the company will be significantly different under different circumstances. We first centralize the independent variable (managers working hours per week) and the reconciliation variable (manager compensation). Secondly, we use the test of three models to analyze step by step whether the salary of management personnel has an intermediary role. Among them, model 1 includes independent variables (workers’ weekly working hours). Model 2 adds moderating variables (manager compensation) based on model 1, and model 3 adds interaction terms (product of manager’s weekly working hours and manager’s compensation) based on model 2.

Table 6 showed the test of the moderating effects of compensation incentives on managers’ working hours and corporate performance. For Model 1, the purpose is to study the influence of independent variable (manager’s weekly working hours) on the dependent variable (enterprise performance) without considering the interference of moderating variable (manager compensation). As can be seen from the above table, the independent variable (manager working hours per week) showed significant significance (t = 2.153, p = 0.045 <0.05). It means that the working hours of managers each week will have a significant impact on the performance of the relationship. However, from the significant results of the interaction term in Model 3, it appears that the moderating role of managerial compensation in the impact of weekly working hours on corporate performance is not significant.

**Table 6:** Test of the moderating effects of compensation incentives on managers’ working hours and corporate performance.

Models and variables	NON-PROFIT ORGANIZATION PERFORMANCE		
	Model 1	Model 2	Model 3
1.Average weekly working hours of managers	0.057	0.045**	0.044*
2.Managerial compensation		0.022*	0.0236**
3.Average weekly working hours of managers×Managerial compensation			0.375
**ΔR^2^**	0.245	0.341	0.034
**R^2^**	0.265	0.486	0.485

### 4.6 Chapter summary

Based on the empirical results, we can draw some important conclusions. First, the average weekly hours worked by managers have a significant positive effect on a nonprofit’s performance, meaning that a larger board and more time commitment can boost an organization’s performance, suggesting that active participation and hard work by board members are critical to an organization’s success. Second, the number of managers on the board is negatively correlated with organizational performance, which means that the size of the board does not necessarily lead to better performance, which requires cooperation and coordination among board members, rather than just increasing the number of people. However, the mediating effect of managers’ remuneration on weekly working hours and organizational performance is not obvious, which may indicate that remuneration is not the key factor affecting managers’ work engagement and organizational performance.

To sum up, the internal governance of non-profit organizations has an important impact on organizational performance, and internal governance factors such as board size, average weekly working hours of members, and salary incentives need to be paid attention to. These results provide useful implications for the management and decision-making of non-profit organizations, and provide important references for future research.

## 5. Discussion

### 5.1 Contributions to theory and practice

This paper’s research has significant theoretical and practical implications and advances the field’s theoretical understanding of non-profit organizations. The study used data from 20 charities’ IRS Form 990 from 2017 in addition to theoretical research on the relationship between internal governance and organizational effectiveness of non-profits. The empirical investigation of the connection between organizational performance and governance is presented in this work. The following are the theoretical and practical contributions of this paper: (i)This study offers a fresh perspective on non-profit organization performance analysis. This article examines the impact of the board of directors and management on the performance of non-profit organizations (non-profit organizations) and examines the regulatory role of compensation incentives (administrative costs) in relation to non-profit organization internal governance and non-profit organization performance; (ii) Our data is more accurate in terms of selection. Previous research frequently used questionnaires and antiquated 990 forms. A new, more transparent Form 990 was designed by the Internal Revenue Service (IRS) in 2007. The 2017 statistics in Table 990 are used in this investigation since they are more precise. It illustrates how American non-profit organization achievement and governance within them are related; (iii)The indicators used in prior organizational performance evaluations are enhanced by our research. This study breaks down several financial figures into percentages: total income minus total expenses; donation income; welfare expenses; total income divided by total assets; investment income split by total income; unrealized investment income divided by total income, etc. evaluating the effectiveness of non-profit institutions. This is currently the center of public attention, particularly in light of the welfare and investment performance of non-profit organizations.

### 5.2 Limitations

Only two of the five hypotheses put out in this work have been confirmed by empirical research findings; the causes and limits are examined as follows: (i) The twenty non-profits chosen for this piece are excessively big. For instance, the biggest NGOs make more than ten times as much money overall as the tiniest non-profits do. This significantly alters some of the data. hence influencing the empirical findings. (ii)The data from the various categories of non-profit organizations likewise varies significantly. Theoretical presumptions and empirical studies will diverge due to variations in the kinds of non-profit organizations. (iii) Because no questionnaires or interviews were used to gather data for this article, more specific information about things like gender, work attitude, and meeting frequency could not be found.

### 5.3 Implications

Through the influence of the above empirical results, some enlightenments are reached as follows: (i)It is suggested that non-profit organizations need to properly arrange the size of the board of directors and the working hours of members to ensure full commitment and enthusiasm for the organizational goals, so as to improve organizational performance. (ii)Warning non-profit organizations to review the composition of the board of directors to avoid problems such as reduced decision-making efficiency and communication efficiency caused by too many people, thus affecting organizational performance. (iii)It implies that the compensation of managers is not the main factor affecting job engagement and organizational performance, and suggests that non-profit organizations should consider other factors, such as work motivation and satisfaction, when setting compensation rewards for managers, rather than simply affecting performance by increasing compensation.

## 6. Conclusion

This paper summarizes in detail the moderating role of compensation incentive in the relationship between internal governance and organizational performance of non-profit organizations. The premise that the internal governance of non-profit organization affects organizational performance is put forward. Through principal component analysis, we find out the three common characteristics that affect the success of non-profit organizations, and calculate the comprehensive score. Through the correlation analysis and regression analysis of the five indicators reflecting the internal governance of non-profit organization, it can be seen that the number of managers is negatively correlated with the performance of non-profit organization, and the average weekly working hours of managers are significantly positively correlated with the performance of non-profit organization. Managers’ compensation is significantly related to the total score of organizational performance. However, the moderating effect between internal governance factors and organizational performance is not significant, which indicates that compensation rewards may not be the key factor leading to changes in internal governance and performance. Therefore, it is of great significance to study the process of the influence of the internal governance structure on the performance of non-profit organization for realizing the organization and sustainable development.

## Supporting information

S1 File The original data was shown in supporting information. (RAR)
